# High-frequency stimulation of the subthalamic nucleus induces a sustained inhibition of serotonergic system via loss of cell phenotype

**DOI:** 10.1038/s41598-022-18294-6

**Published:** 2022-08-17

**Authors:** Faisal Alosaimi, Yasin Temel, Sarah Hescham, Victoria S. Witzig, Faris Almasabi, Sonny K. H. Tan, Ali Jahanshahi

**Affiliations:** 1 Department of Neurosurgery, Maastricht University Medical Center, P. Debyelaan 25, 6202AZ Maastricht, The Netherlands; 2 Department of Neurology, University Hospital RWTH Aachen, Aachen, Germany; 3 Department of Neurosurgery, University Hospital RWTH Aachen, Aachen, Germany

**Keywords:** Diseases of the nervous system, Neural circuits, Neuronal physiology

## Abstract

Deep brain stimulation (DBS) of the subthalamic nucleus (STN) has become a standard treatment for Parkinson’s disease (PD). However, in a considerable number of patients debilitating psychiatric side-effects occur. Recent research has revealed that external stimuli can alter the neurotransmitters’ homeostasis in neurons, which is known as “neurotransmitter respecification”. Herein, we addressed if neurotransmitter respecification could be a mechanism by which DBS suppresses the serotonergic function in the dorsal raphe nucleus (DRN) leading to mood changes. We infused transgenic 5-HT-Cre (ePET-Cre) mice with AAV viruses to achieve targeted expression of eYFP and the genetically encoded calcium indicator GCaMP6s in the DRN prior to methyl-4phenyl-1,2,3,6-tetrahydropyridine (MPTP) treatment. Mice received bilateral DBS electrodes in the STN and an optic fiber in the DRN for calcium photometry. MPTP-treated mice demonstrated behavioral and histological PD phenotype, whereas all STN-DBS animals exhibited an increased immobility time in the forced swim test, reduced calcium activity, and loss of tryptophan hydroxylase-2 expression in the DRN. Given the prominent role of calcium transients in mediating neurotransmitter respecification, these results suggest a loss of serotonergic phenotype in the DRN following STN-DBS. These findings indicate that loss of serotonergic cell phenotype may underlie the unwanted depressive symptoms following STN-DBS.

## Introduction

Deep brain stimulation (DBS) has emerged as a successful neurosurgical treatment to treat selected neurological and psychiatric disorders^1–4^. DBS of the subthalamic nucleus (STN) has particularly shown to effectively improve medically intractable motor symptoms of Parkinson’s disease (PD)^5–8^. Despite long-term improvement in motor function, some PD patients exhibit mood disorders such as depression, suicide ideation and impulsivity after surgery^9,10^.

Our earlier studies have shown that acute bilateral STN-DBS inhibits neurotransmission of the midbrain serotonin (5-hydroxytryptamine; 5-HT) system in the dorsal raphe nucleus (DRN), which is the main source of 5-HT in the central nervous system and its dysfunction has been associated with the onset of mood disorders^11^. Acute STN-DBS in experimental animal studies demonstrated reduced firing rate of DRN 5-HT neurons, decreased 5-HT release in the forebrain and induction of depressive-like behavior in PD rats^12,13^. However, in clinical settings STN-DBS is applied chronically. Long-term modulation of neuronal networks may induce permanent and neuroplastic changes^14^. More recently, it has been demonstrated that neurotransmitter identity in the mature brain can be influenced by environmental stimuli^15^. Neurotransmitter switching, induction or elimination associated with altered behavioral output are termed neurotransmitter respecification^16–18^. We hypothesized that neurotransmitter respecification plays a role in STN-DBS and occurs in the DRN 5-HT system. To investigate this we used the transgenic mouse line expressing Cre under the enhancer of the transcription factor Pet1 (ePET-Cre), which allows selective targeting of DRN 5-HT neurons^19^. These transgenic mice with PD associated symptoms after methyl-4phenyl-1,2,3,6-tetrahydropyridine (MPTP) administration were treated with daily STN-DBS for a relatively long period of time compared to existing studies. Behavioral, photometric and immunohistochemical assessments were used to evaluate aspects of neurotransmitter respecification in the DRN 5-HT system.

## Results

Stimulating electrodes were positioned bilaterally and symmetrically (inter-electrode variation < 0.1 mm) in the STN in all mice except two, for which electrodes were located in the zona incerta. Those mice were excluded from the analysis. An example of electrode trajectory in a coronal brain section, and location of all electrode tips in the STN map are shown in the supplementary material (Fig. S1A-B). Fiber photometry probes were placed in the dorsomedial segment of the DRN in all mice except three, which were excluded from signal processing. No signs of significant histological damage due to implantation or electrical stimulation were observed.

**Figure 1:**
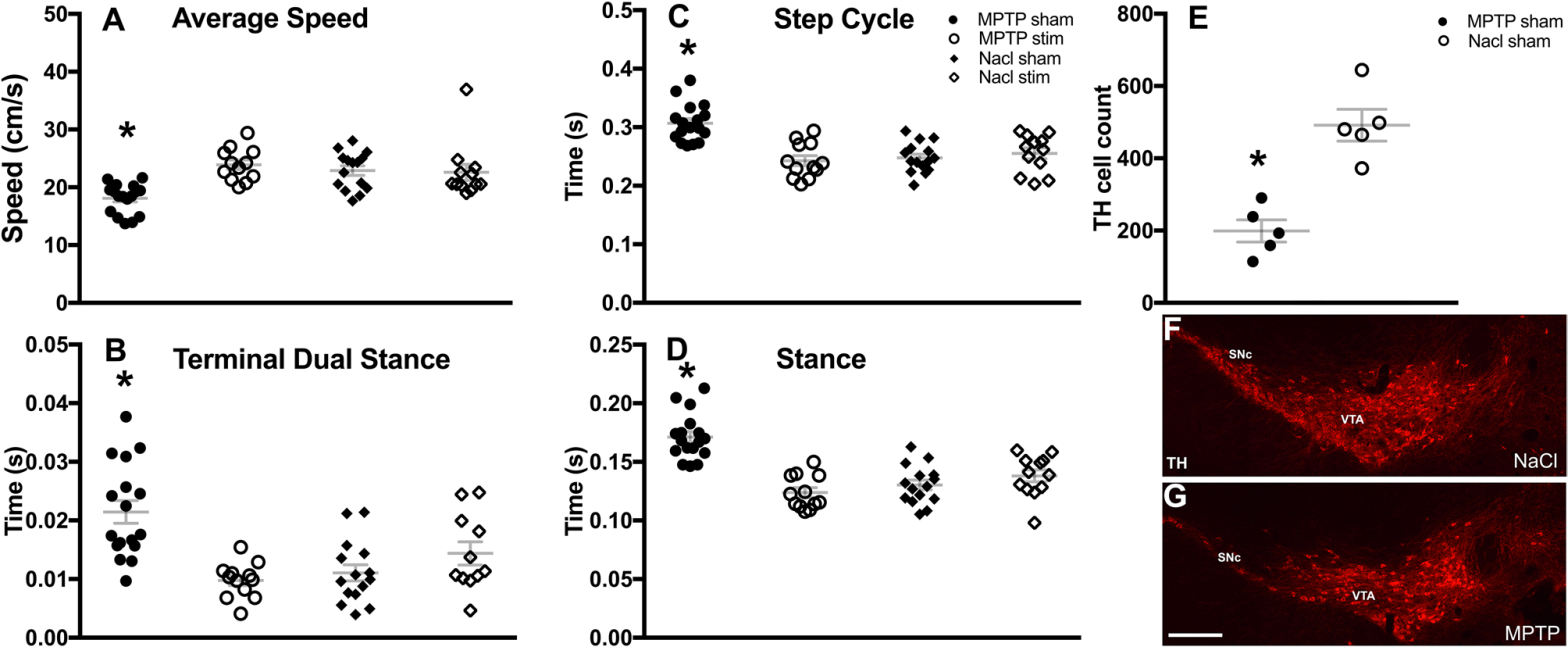
Effect of MPTP treatment and intermittent STN-DBS on Catwalk dynamic and static gait parameters. (**A**–**D**) Graphs show a significant reduction in speed, and increases of step cycle, terminal dual stance and stance in MPTP-sham mice. STN-DBS restored those parameters to control levels, which is indicated by non-significant differences between MPTP-stim and NaCl-sham groups, and significant differences between MPTP-stim and MPTP-sham groups. (**E**) The graph shows a significant reduction in TH positive cells in the SNc of MPTP-treated mice compared to the NaCl-treated animals. (**F**–**G**) Representative low-power photomicrograph of coronal brain sections containing the SNc and VTA, stained for TH, show a noticeable TH cell loss in MPTP vs NaCl-treated mice. Data are presented as mean + /− SEM; significant difference (*P* < 0.05) is indicated by a “*”, scale bar = 250 µm. Tyrosine hydroxylase, TH; substantia nigra pars-compacta, SNc; ventral tegmental area, VTA; subthalamic nucleus, STN; methyl-4phenyl-1,2,3,6-tetrahydropyridine, MPTP; deep brain stimulation, DBS.

MPTP-treated mice showed a PD-like motor phenotype compared to the NaCl-treated group. MPTP treatment induced significant static and dynamic gait impairments with reduced average speed [MPTP-sham: 18.09 ± 0.62; MPTP-stim: 23.91 ± 0.68; NaCl-sham: 22.90 ± 0.80, and NaCl-stim: 22.60 ± 1.17; Two-way ANOVA; group effect: F(3,52) = 9.04, *p* < 0.001; disease*group effect: F(1,52) = 3.64, *p* < 0.001; followed by Bonferroni pairwise comparison; MPTP-sham vs NaCl-sham: *p* < 0.001; Fig. 1A], increased terminal dual stance [MPTP-sham: 0.021 ± 0.002; MPTP-stim: 0.009 ± 0.001 NaCl-sham: 0.011 ± 0.001, and NaCl-stim: 0.015 ± 0.002; Two-way ANOVA; group effect: F(3,52) = 10.36, *p* < 0.001; disease*group effect: F(1,52) = 2.90, p = 0.160; followed by Bonferroni pairwise comparison; MPTP-sham vs NaCl-sham: *p* < 0.001; Fig. 1B], step cycle [MPTP-sham: 0.31 ± 0.008, MPTP-stim: 0.24 ± 0.007; NaCl-sham: 0.25 ± 0.006, and NaCl-stim: 0.26 ± 0.008; Two-way ANOVA; group effect: F(3,52) = 15.28, *p* < 0.001; disease*group effect: F(1,52) = 8.30, *p* < 0.01; followed by Bonferroni pairwise comparison; MPTP-sham vs NaCl-sham: *p* < 0.001; Fig. 1C], and stance [MPTP-sham: 0.17 ± 0.005, MPTP-stim: 0.12 ± 0.004; NaCl-sham: 0.13 ± 0.004, and NaCl-stim: 0.14 ± 0.004; Two-way ANOVA; group effect: F(3,52) = 23.08, *p* < 0.001, disease*group effect: F(1,52) = 8.17, *p* < 0.001; followed by Bonferroni pairwise comparison; MPTP-sham versus NaCl-sham: *p* < 0.01; Fig. 1D].

Moreover, STN-DBS restored these gait parameters in MPTP-treated mice with a significant increase in average speed and decrease in terminal dual stance, step cycle and stance [Two-way ANOVA; group effects: F(3,52) = 9.04, *p* < 0.001; F(3,52) = 10.36, *p* < 0.001; F(3,52) = 15.28, *p* < 0.001; and F(3,52) = 23.08, *p* < 0.001, respectively; stim*group effects: F(1,52) = 9.07, *p* = 0.064; F(1,52) = 4.75, *p* < 0.05; F(1,52) = 12.02, *p* < 0.01, and F(1,52) = 17.95, *p* < 0.01, respectively]. Bonferroni post-hoc pairwise comparison of the means showed significant differences between MPTP-sham vs MPTP-stim in all tests (*p*’s < 0.001; Fig. 1A–D). Furthermore, stimulation did not alter gait parameters in NaCl-treated mice (NaCl-sham vs NaCl-stim) in none of the tests (Bonferroni post-hoc pairwise comparison: *p*’s > 0.05). Post-mortem TH-immunohistochemistry revealed a significant loss (average 60%) of SNc dopaminergic neurons after MPTP administration in comparison to NaCl treatment (MPTP-sham: 198.8 ± 30.54 vs. NaCl-sham: 491.8 ± 43.82; independent samples T-test *p* < 0.005; Fig. 1E–G).

**Figure 2:**
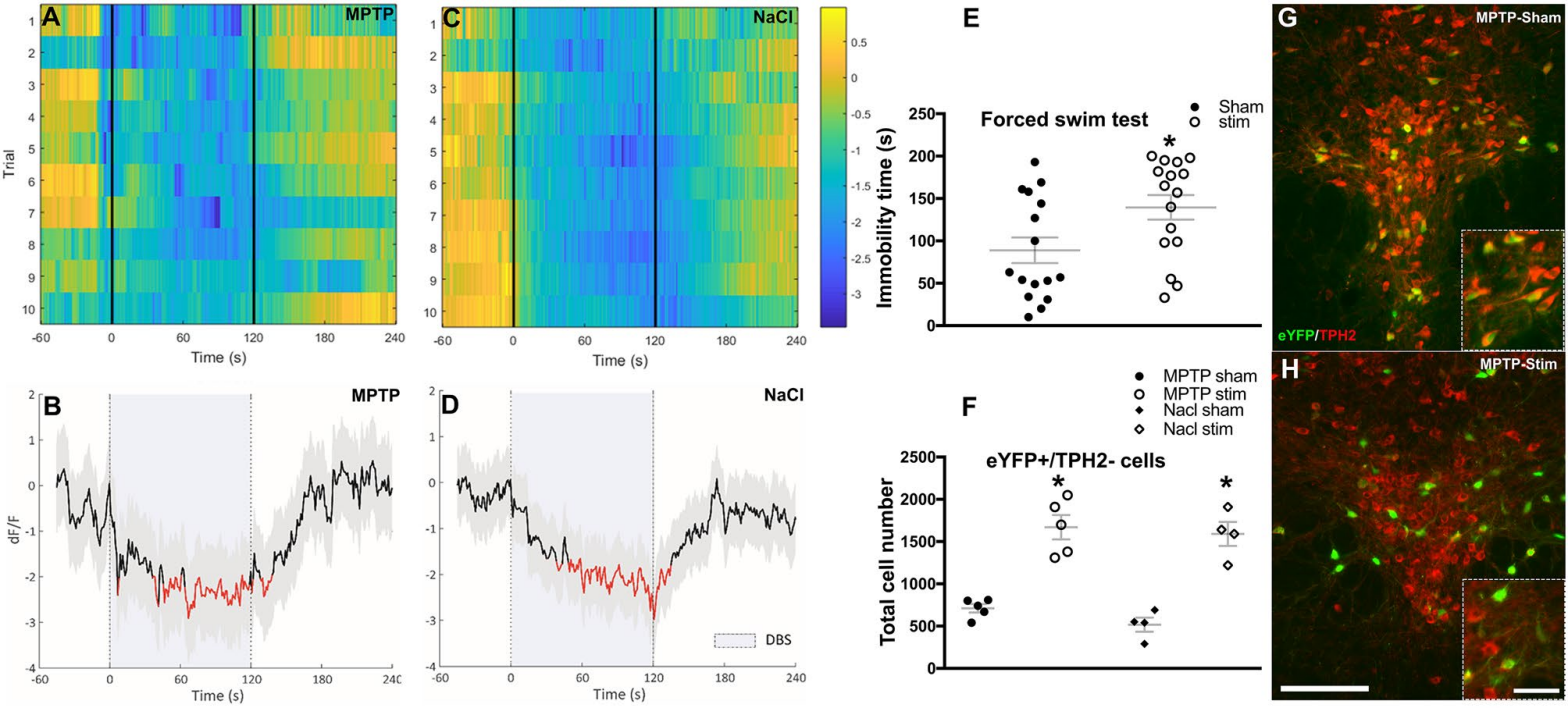
Effect of STN-DBS on serotonergic system. The effect of STN-DBS on activity of 5-HT neurons in the DRN measured with genetically coded calcium sensor GCaMP6s (fiber photometry). (**A**) and (**C**) examples of heat-maps of the change in fluorescence (dF/F) before, during (the stimulation period is indicated by vertical lines), and after DBS in MPTP and NaCl-treated mice, respectively. Each row plots one DBS session (total of 10 trials). Color scale at the right indicates dF/F (yellow = high and dark blue = low dF/F). (**B**) and (**D**) the bottom plots show the cumulative changes in fluorescence averaged over the ten trials in MPTP- (*n* = 14) and NaCl-treated mice (*n* = 17). The thick black line indicates mean, shaded areas indicate SEM, and red segments indicate statistically significant decrease from baseline (DBS period is indicated by vertical dashed lines; *p* < 0.05; permutation test). (**E**) STN-DBS induced depressive like-behavior in forced swim test, shown by an increased immobility time in stimulated animals. (**F**) The graph shows that chronic STN-DBS significantly reduced TPH2 expression in transfected (eYFP expressing) cells in both MPTP and NaCl-treated mice. (**G**), (**H**) representative photomicrographs of coronal brain sections containing the DRN display eYFP expressing cells (green) that were double labelled with antibody raised against TPH2 (red; scale bar = 150 µm). Insets in (**G**, **H**) show higher magnification of eYFP cells that with and without TPH2 labeling (Scale bar = 50 µm). Data are presented as mean + /− SEM; significant difference (*P* < 0.05) is indicated by a “*”. Subthalamic nucleus, STN, dorsal raphe nucleus, DRN, enhanced yellow fluorescent protein, eYFP; Tryptophan hydroxylase-2, TPH2; methyl-4phenyl-1,2,3,6-tetrahydropyridine, MPTP; deep brain stimulation, DBS.

Fiber photometry assessing calcium transients of DRN neurons showed a significant reduction of GCaMP6s fluorescence, indicating neuronal inhibition upon STN-DBS (Fig. 2A–D). Permutation test showed decreased calcium signaling by STN-DBS in both MPTP and NaCl-treated mice (*p* < 0.05). After stimulation was halted GcaMP6s fluorescence signal returned to baseline within ninety seconds.

FST test was discontinued due to the risk of drowning. As a result comparing four groups with an ANOVA test was not possible due to the low sample size. Instead, the data of stimulated animals (NaCl-stim and MPTP-stim) were pooled compared to the sham (NaCl-sham and MPTP-sham) animals (stim: 139.56 ± 14.39 vs sham: 88.93 ± 15.13; independent samples T-test, *p* < 0.05; Fig. 2E). STN-DBS induced behavioral despair in the FST, which was apparent by increased immobility time in comparison to non-stimulated mice. This depressive-like behavior after STN-DBS was observed in both MPTP and NaCl-treated mice.

After we established that STN-DBS induced depressive-like behavior and decreased calcium signaling in the DRN, we subsequently evaluated the phenotype of genetically targeted DRN 5-HT neurons. Stereological cell counts of double-labelled enhanced yellow fluorescent protein (eYFP)/tryptophan hydroxylase-2 (TPH2) expressing neurons in the DRN showed a significant increase of eYFP positive/TPH2 negative neurons in STN-DBS treated mice in comparison to sham stimulated animals [MPTP and NaCl-stim: 1670 ± 144 and 1590 ± 141, vs MPTP and NaCl-sham: 712 ± 50 and 518 ± 83, respectively; Two-way ANOVA; group effect: F(3,14) = 27.60, *p* < 0.001; disease*group effect: F(1,14) = 1.48, *p* = 0.24; and stim*group effect: F(1,14) = 81.08, *p* < 0.001; Fig. 2F–H]. This inhibitory effect of chronic STN-DBS on TPH2 expression was found to be independent of the integrity of the nigrostriatal dopamine pathway as this observation was present in both MPTP and NaCl-treated mice when tested by Bonferroni post-hoc pairwise comparison of the means [MPTP-sham vs MPTP-stim: *p* < 0.001; NaCl-sham vs NaCl-stim: *p* < 0.001; MPTP-sham vs NaCl-sham: *p* = 1.00 and MPTP-stim vs NaCl-stim: *p* = 1.00; Fig. 2F]. Moreover, neither STN-DBS nor MPTP administration altered neuronal c-Fos expression in the DRN, and no significant changes between groups were found [Two-way ANOVA; group effect: F(3,18) = 0.31, *p* = 0.81; Fig.S3]. Finally, quantification of TPH2 and eYFP expressing cells in the DRN did not reveal any significant difference between groups [Two-way ANOVA; group effects: (F(3,14) = 0.40, *p* = 0.76; and F(3,14) = 1.73, *p* = 0.21, respectively; Fig. S4).

## Discussion

In this study, we investigated the neuroplastic effects of DBS on neurotransmitter phenotype. Recently, neurotransmitter respecification in the adult brain was described in which external cues induced neurotransmitter phenotype switching, neurotransmitter induction or elimination with concurrent behavioral alterations^16–18^. We hypothesized that this phenomenon might play a role in DBS. This may be particularly relevant for STN-DBS as a widely accepted neurosurgical treatment in medically refractory PD with stimulation-dependent motor and non-motor behavioral changes^5–8^. Patients may experience depressive symptoms after surgery, which by itself is a risk factor for post-operative suicide^9,10^. Understanding the neuronal mechanisms of these behavioral changes is relevant for the more than 208,000 patients that are already treated by DBS worldwide^20^.

We used the ePET-Cre mouse line, which enables a specific assessment of 5-HT neurons in the DRN synthesizing TPH^19^. MPTP administration in these mice resulted in a significant loss (approximately 60%; Fig. 1E) of SNc dopamine neurons and displayed gait impairments that were alleviated by STN-DBS, overall mimicking dopaminergic degeneration and beneficial motor effects of stimulation in PD patients (Fig. 1A–D).

In addition, STN-DBS elicited behavioral despair in MPTP mice, which is considered as reflecting depressive-like behavior (Fig. 2E). This behavioral change by STN-DBS was independent of the integrity of the nigrostriatal pathway and motor function as NaCl-treated mice showed similar behavioral output (Fig. S2). This observation was also reported by our previous studies^12,21^. Pretreatment with the selective-serotonin reuptake inhibitor citalopram before STN-DBS was effective in preventing behavioral despair^12^. This pinpointed towards a 5-HT-dependent mechanism and triggered experiments investigating downstream effects of STN-DBS to the brainstem 5-HT system, with the DRN as the major source of 5-HT innervation to the forebrain^11^.

Using fiber photometric measurements of calcium signaling, we demonstrated in this study that intermittent STN-DBS decreased calcium signaling and caused neuronal inhibition within the DRN (Fig. 2A–D). This is in line with acute STN-DBS electrophysiological experiments where stimulation decreased 5-HT neuronal firing rate by 40–50 in extra-cellular single cell recordings^12,22^. Subsequent in vivo microdialysis experiments also found decreased 5-HT release in terminal forebrain regions as expected^13,23^. Previous studies have focused on the underlying neuronal circuit. Since STN projecting neurons to the DRN are lacking it has been postulated that inhibition of 5-HT neurotransmission is mediated by a multi-synaptic neuronal network. The lateral habenula may contribute to this network as a well-defined major inhibitory input structure to the DRN and has been attributed a critical role in 5-HT feedback mechanisms^21,22^. Although STN receives 5-HT inputs from the DRN, there is no evidence regarding direct effect of STN-DBS on 5-HT cells via these inputs. Electrophysiological studies have demonstrated that STN-DBS did not induce antidromic or short-latency (< 10 ms) orthodromic responses in peristimulus time histograms recorded from the DRN^12^. We also found that STN-DBS increased neuronal activity with c-Fos expression in the lateral wings of the DRN, which receive major input from various forebrain regions, including the lateral habenula^21^. However, other mechanisms such as 5-HT receptor mediated inhibition or changes in DRN microcircuitry cannot be completely ruled out and may contribute to our observations. Acute STN-DBS has been shown to alter neuronal firing rates of habenular neurons projecting to the DRN^22^. It remains undetermined how STN-DBS influences 5-HT neurotransmission and homeostasis. It has been shown, however, that some cells regain the ability to fire intrinsic spikes of action potential in the presence of continuous stimulation^24^, whereas other neurons remain inhibited after cessation of stimulation^22^. These altered activities most likely influence stringent 5-HT feedback mechanisms and may trigger neuroplasticity within the network.

Our earlier study indicated that DBS of the anterior nucleus of the thalamus increased the number of dopaminergic neurons in the ventral tegmental area^25^. This might have been indicative for DBS induced neurotransmitter respecification. In the current study eYFP positive neurons in the DRN should typically express TPH2 in the vast majority (> 90%)^19^. Interestingly, we found STN-DBS to reduce the number of double-labelled eYFP/TPH2 positive neurons quantified by stereological methods (Fig. 2F–H). Although ePET-Cre genetically targets DRN 5-HT neurons specifically, it should be kept in mind that it represents a part of the total 5-HT population^19^. Moreover, 5-HT cells only around the infusion site were transfected in this study. Stereological quantification of eYFP and TPH2 expressing cells in the DRN revealed no significant difference between groups (Fig. S4). In addition, c-Fos expression in the DRN was not altered by STN-DBS, suggesting that overall neuronal activity after intermittent stimulation remained stable (Fig. S3).

Activity-dependent intracellular calcium transients play a key role in neurotransmitter respecification by regulating the phosphorylation of transcription factors that are critical in defining the neurotransmitter phenotype of cells^17,26,27^. However, how calcium transients alter neurotransmitter respecification, seems to differ across transmitter systems and species. For instance, elevated activity of dopaminergic neurons in the paraventricular nucleus of the hypothalamus in adult rats was shown to be required for the loss of dopamine expression after long-day photoperiod exposure^18^. Whereas, decreases in calcium spiking by exposure of Xenopus laevis to dark lead to loss of dopamine expression in the hypothalamus^28^. Seemingly, altered calcium transients could lead to opposite effects in serotonergic neurons. Suppression of activity in the Xenopus laevis hindbrain generated an increase in the number of neurons expressing TPH in the raphe nucleus. Whereas, enhancement of activity led to the opposite result^27^. In our study, a decrease in the number of TPH2 expressing neurons was associated with reduced Ca2 + transients. This contrasts with respecification of dopaminergic cells in rats^18^, in which an increase in Ca2 + activity correlated with the loss of dopaminergic cell phenotype. It should be noted that the extent to which the 5-HT cells were transfected with the GCaMP6s virus was not quantified in this study. Therefore, it is plausible that Ca2 + transients were not measured in all serotonergic cells.

Initial theories suggested that DBS at stimulation settings commonly used in clinical practice decreases spontaneous firing of neuronal populations and drives axonal projections near the electrode also known as “firing rate model” which was based on real-time and local effects of DBS^14^. Nowadays, ample evidence show that changes in neuronal activity per se are unsustainable states, and neurons regain their intrinsic activity overtime^24^ and electrical stimulation results in prolonged plasticity-associated effects even when stimulation is turned off^29^. Similarly, transient changes in Ca2 + activity could lead to transmitter respecification, which can have a network and biochemical effects that transcend the time of stimulation. Altogether these behavioral, photometric and immunohistochemical data pinpoint to a key role for stimulus-derived loss of 5-HT cell phenotype. We argue that this loss of 5-HT phenotype plays a key role in unwanted depressive symptoms following STN-DBS. The fade of 5-HT phenotype could also be the mechanism whereby STN-DBS reduces treatment-resistant tardive dyskinesia. The 5-HT system has been implicated in the symptoms of dyskinesia. Extensive 5-HT innervation of the basal ganglia modulates dopamine neurotransmission^30,31^. The lower incidence of dyskinesia is associated with 5-HT2 receptor antagonism^32,33^. Moreover, symptoms of dyskinesia can be exacerbated by concomitant treatment with selective serotonin reuptake inhibitors^34–36^. Based on our observation that STN-DBS suppresses 5-HT cell phenotype, one may conclude that reduction in basal ganglia 5-HT function is a key component of the DBS therapeutic mechanism in dyskinesia.

In conclusion, understanding neuroplastic effects is critical to our understanding of network modulation by DBS and symptom reduction or side effects. This study reveals evidence that STN-DBS induces changes in calcium signaling in the midbrain raphe nuclei 5-HT system and results in neurotransmitter respecification, which may play a role in psychiatric side effects in PD. The loss of 5-HT cell phenotype could also be the mechanism whereby STN-DBS reduces treatment-resistant tardive dyskinesia.

## Methods

### Animals

Experiments were performed on 56 male transgenic ePET-Cre mice (JAX stock; #012,712). Animals were socially housed under constant temperature, humidity and reversed dark/light cycle (12 h each) with free access to food and water. All animal procedures were performed in accordance with “Animal Research: Reporting of in vivo Experiments (ARRIVE)” guidelines. Animal procedures were reviewed and approved by the Institutional Animal Care Committee of Maastricht University in accordance with the Central Authority for Scientific Procedures on Animals (CCD; protocol # AVD107002016543).

### Induction of Parkinson’s disease model and stereotactic surgery

Mice were randomly assigned into one of the following four groups: NaCl-sham, NaCl- STN-DBS, MPTP-sham or MPTP-STN-DBS. Mice were injected with MPTP (30 mg/Kg i.p) or NaCl (0.9% i.p.) for five consecutive days, two weeks prior to stereotactic surgery. Stereotactic surgery^37^ was performed under isoflurane inhalation anesthesia (Abbott Laboratories; induction 4%, maintenance 1.5–2%) after analgesic pretreatment (buprenorphine, 0.1 mg/Kg s.c). The mouse head was positioned and fixated in a stereotaxic frame (Stoelting). A body temperature of 37 °C was maintained with a thermo-regulator pad. After local anesthesia (lidocaine 1% s.c.), the skull was exposed and burr holes were made for implantation of bilateral STN electrodes (coordinates from bregma based on mouse brain atlas: AP − 2.00 mm, ML ± 1.50 mm, DV − 4.55 mm ^38^) and a fiber photometry probe (400 μm; 0.48NA Patchcord) was implanted in the DRN (coordinates from bregma based on mouse brain atlas: AP − 4.5, ML − 0.25, DV-2.9 at a 32° angle from the left).

### Viral transfection

During the same surgery and before implantation took place, two viral vectors were injected into the DRN. A Cre-dependent adeno-associated virus encoding for eYFP (AAV5.EF1a.DIO.eYFP.WPRE.hGH; Penn Vector Core, USA) was injected (1.0 µl, at a rate of 0.1 μl/min) into the DRN. In addition, an AAV vector ensuring targeted genetic encoding of the fluorescent Ca2 + indicator GCaMP6s (AAV5.Syn.Flex.GCaMP6s.WPRE.SV40; Addgene, USA) was also injected into the same coordinates (500 nL; Nanoject I; Drummond Scientific).

### Deep brain stimulation

After 2 weeks of recovery, STN-DBS was performed for 10 weeks with 20 min stimulation sessions (5 times a week) with monophasic high-frequency stimulation at 130 Hz, a pulse width of 60 μs and a current intensity of 80 μA. Sham stimulated animals were connected but stimulation was omitted. The DBS construct consisted of two bipolar gold-coated concentric electrodes, with interelectrode distance of 3.0 mm and 5.5 mm length each. The outer stainless steel and inner platinum-iridium parts function as the positive and negative poles, respectively. The outer diameter of the concentric needle is 300 μm (including the insulation), the electrode surface is 0.021 mm^2^, and the distance between anode and cathode is 50 μm^37^. The surface of the electrode is 0.021 mm^2^, so the chosen parameters resulted in a charge density of 22.9 μC/cm^2^, which is well below the limit of 30 μC/cm^2^ based on the Shannon model of neuronal damage^39^.

### Fiber photometry

Ca2 + transients of DRN neurons were measured in MPTP and saline-treated mice using an established fiber photometry technique^37^. This method enabled measuring the bulk Ca2 + -dependent fluorescence of GCaMP6 during STN-DBS. A two-wavelength GCaMP fiber photometry system (Doric Lenses Inc., Quebec, Canada) was utilized for calcium signal recording. GCaMP and Ca2 + -independent fluorescent signals were alternatingly excited by a 470 nm LED and a 405 nm LED (isosbestic reference signal), respectively. GCaMP6s fluorescence emissions were captured with a Newport 2151 Femtowatt Photoreceiver Module and the signals relayed into a Field Programmable Gate Array (FPGA)-based data acquisition unit which integrates with the Doric Neuroscience Studio software. During the photometry experiment, mice could move freely in their home cage. STN-DBS was applied intermittently (2 min on − 3 min off) for ten trials (5 min per trial) during which photometry measurements were performed in the DBS on/off phases. We extracted, processed and analyzed the calcium transients with a custom MATLAB (Mathworks) script. The first 2.5 min of the data during the habituation period were discarded to remove the initial fast bleaching of the fluorescent signal. Next, the original sampling rate of a 100 Hz was downsampled to 1 Hz and low-pass filtered. A two-term exponential model was fitted and subtracted from the decimated data to account for slow bleaching artifacts. Then, a single baseline fluorescence value (F0) was calculated by averaging the fluorescent signals during the 60-s time period pre-DBS. Subsequently, the normalized change in fluorescence (dF/F) was calculated as F − F0/F0. Data are presented as an average plot with SEM. A permutation test was used to analyze the statistical significance of the DBS-related fluorescent change^40^. To compare the values of dF/F at each time point with the DBS-related fluorescent change, 10,000 permutations were used. An α-level of ≤ 0.05 was considered significant.

### Behavioral assessment

#### Gait analysis

MPTP and STN-DBS related motor effects were assessed by a computerized gait analysis setup (CatWalkXT; Noldus). Mice ran through an enclosed corridor with a hard glass-plated floor. Footprints were recorded by a high-speed camera from which gait-related movement parameters were analyzed, including average speed, step cycle, terminal dual stance and stance. Five consecutive uninterrupted straight runs of each mouse were used for statistical analysis^41^.

#### Forced swim test

The forced swim test (FST) was used to evaluate despair behavior based on a published protocol^42^. Mice were placed in an inescapable plastic cylindrical container (height 40 cm × diameter 19 cm) filled with a 23–25 °C water (30 cm deep). The duration of immobility was recorded during a trial of 6 min. Immobility was defined as the time of not moving or with slight movements to keep the nose above the water surface.

### Tissue processing and immunohistochemistry

At the end of the experiments, mice were deeply anaesthetized with pentobarbital and transcardially perfused with tyrode buffer, followed by ice-cold 4% paraformaldehyde fixative in 0.1 M phosphate buffer. The brains were extracted, fixed in 4% paraformaldehyde overnight and submerged in 20% sucrose for 24 h at 5 °C. The brains were sectioned in coronal slices (thickness: 22 µm) on a cryostat and stored at -80 °C. A standard hematoxylin–eosin staining was performed to assess the electrode tip location (Fig. S1 A). Animals with misplaced electrodes were excluded from behavioral and histological analysis.

#### Tyrosine hydroxylase immunohistochemistry

MPTP-induced dopamine depletion was evaluated by tyrosine hydroxylase (TH) immunohistochemistry. Sections containing the SNc were incubated overnight with a primary antibody raised against TH (rabbit polyclonal anti-TH antibody; Santa Cruz Biotechnology Inc; 1:1000). On the next day, sections were incubated with a secondary antibody (donkey anti-rabbit alexa 647, Jackson Immunoresearch Laboratories; 1:400) for one hour. Thereafter, the sections were mounted and coverslipped (Immu-Mount, USA). Photographs of two anatomical bregma levels (coordinated based on mouse brain atlas AP − 2.92 and − 3.16 ^38^) were taken with an Olympus DP70 digital camera connected to an Olympus BX50 microscope. A semi-quantitative TH cell count was performed using ImageJ software (National Institutes of Health, USA).

#### c-Fos immunohistochemistry

To assess overall neuronal activity of the DRN immunohistochemical expression of c-Fos was evaluated. The DRN sections were incubated overnight with a primary anti-c-Fos antibody (rabbit polyclonal anti-c-Fos; Abcam; 1:1000). This was followed by incubation for one hour with a secondary antibody (donkey anti-rabbit alexa 594, Jackson immunoresearch Laboratory; 1:200). Eight slices were selected from bregma − 4.16 to − 4.96 and photographed with an Olympus BX51 fluorescence microscope (Olympus, Germany) connected to an Olympus Camera DP72 (Olympus, Germany). All clear c-Fos expressing neurons were counted (FiJi v2.0.0, National Institutes of Health; Maryland).

#### Tryptophan hydroxylase-2 immunohistochemistry

To assess whether STN-DBS influenced 5-HT synthesis of eYFP expressing 5-HT DRN neurons, tissue was processed for TPH2 immunohistochemistry, which is the rate-limiting enzyme in 5-HT synthesis. DRN sections were incubated overnight with a primary anti-TPH2 antibody (goat polyclonal anti-TPH2; Abcam; 1:2000). This was followed by incubation with a secondary antibody (donkey anti-goat alexa 647, Jackson Immunoresearch Laboratories; 1:200) for two hours. Stereological analysis of double-labelled eYFP/TPH2 neurons was performed (Stereo Investigator, Microbrightfield Bioscience, Williston, VT, USA) in seven DRN sections per mouse using a fluorescence spinning disk confocal microscope (DSU, Olympus BX51, Japan) connected to a digital ultra-high sensitivity CCD camera (C9100-02, Hamamatsu Photonics, Japan). Stereological cell counting was performed using the optical fractionator probe and total double-labelled cell number was estimated using a validated stereological method^43,44^.

### Data analysis

Statistical analysis was performed using SPSS 26.0 software (SPSS Inc., Chicago, USA). Behavioral and immunohistochemical data were analyzed using two-way ANOVA. Bonferroni post-hoc pairwise comparison was conducted, if (and only if) the global ANOVA test result was significant. To compare the two-groups’ data, we used an independent T-test. Data are presented as mean values and standard error of means (± SEM). All data were normally distributed, and statistical significance was defined by a *p*-value < 0.05. Photometry data was processed and analyzed with custom Matlab (MathWorks) scripts. A permutation test was performed to statistically evaluate calcium transients^40^.

## Supplementary Information

Supplementary Information.
