# Supplementary material for: High-frequency stimulation of the subthalamic nucleus induces a sustained inhibition of serotonergic system via loss of cell phenotype
Source: Sci Rep. 2022 Aug 17;12:14011. doi: 10.1038/s41598-022-18294-6 (PMC9385659; doi:10.1038/s41598-022-18294-6)
Supplement: Supplementary file 1 — Supplementary Information. [file 41598_2022_18294_MOESM1_ESM.docx]

**Title: High-frequency stimulation of the subthalamic nucleus induces a sustained inhibition of serotonergic system via loss of cell phenotype**

Faisal Alosaimi^1,2^, Yasin Temel^1^, Sarah Hescham^1^, Victoria S. Witzig^3^, Faris Almasabi ^1,5^, Sonny K. Tan^1,4^ and Ali Jahanshahi^1*^

*
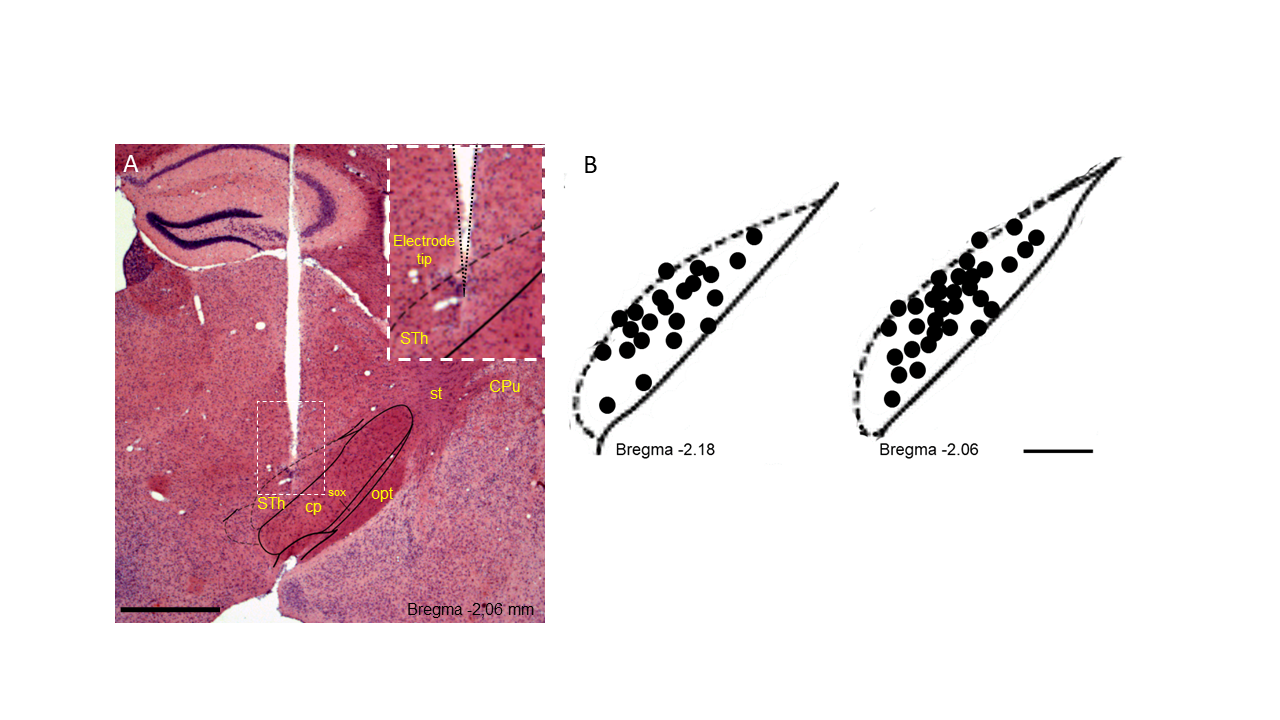
***Supplementary figures:**

Figure 1: A) Representative low-power photomicrograph of coronal brain section stained with Hematoxylin and Eosin (H&E) shows the deep brain stimulation electrode tip at the subthalamic nucleus (STN, scale bar=500µm). B) Illustrative coronal images showing 54 electrode tip locations in or close to the STN, as verified by post hoc histology (Scale bar=200µm). STh, subthalamic nucleus; cp, cerebral peduncle; CPu, caudate-putamen; sox, supraoptic decussation; opt, optic nerve; st, stria terminalis.


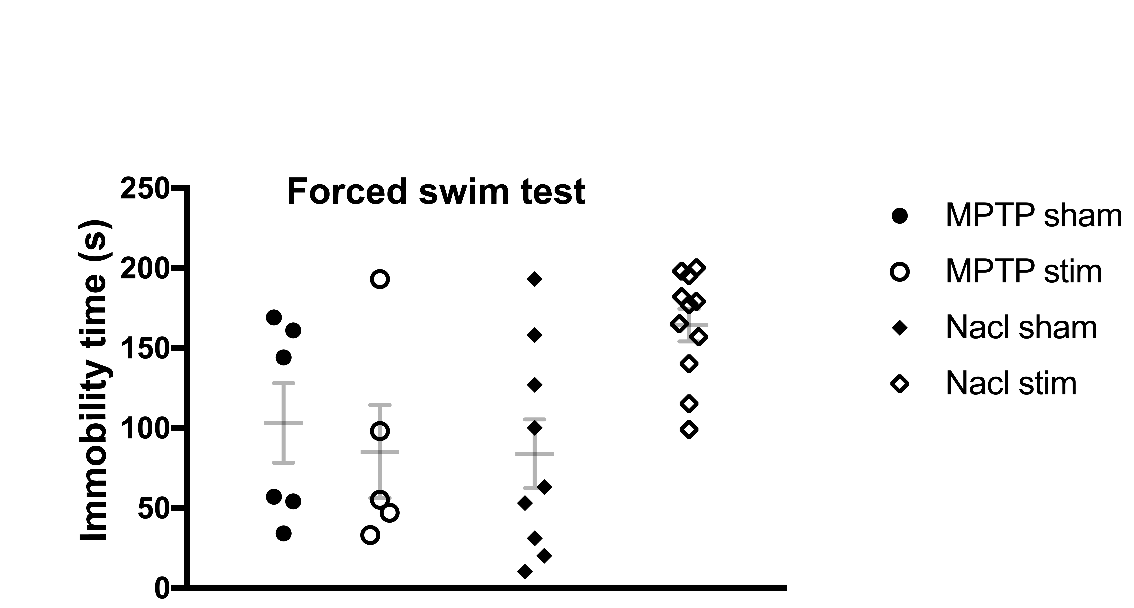


Figure 2: **Effect of STN-DBS on depressive-like behavior.**

Graph shows the quantification of immobility time of mice in the forced swim test. Statistical analysis revealed no significant difference between groups (F (3,27)=4.56, p=0.11,two-way ANOVA). Data are presented as mean +/- SEM.


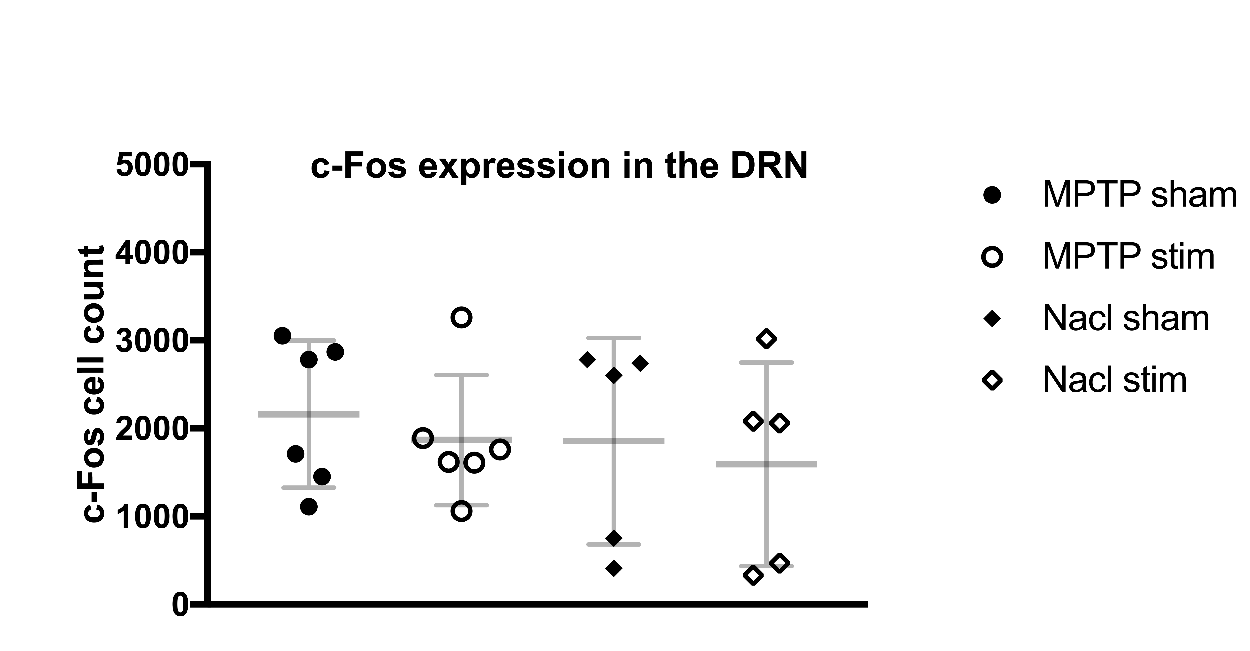


Figure 3: **Effect of STN-DBS on the overall neuronal activity in the DRN**.

Graph shows the quantification of c-Fos positive cells in the dorsal raphe nucleus (DRN) of mice. Statistical analysis revealed no significant difference between groups (F(3,18)=0.31, p=0.81,two-way ANOVA; F (1,18) =0.001 p=0.97). Comparison between stimulated and non-stimulated mice did not reveal a significant difference (independent samples T-test p=0.48).


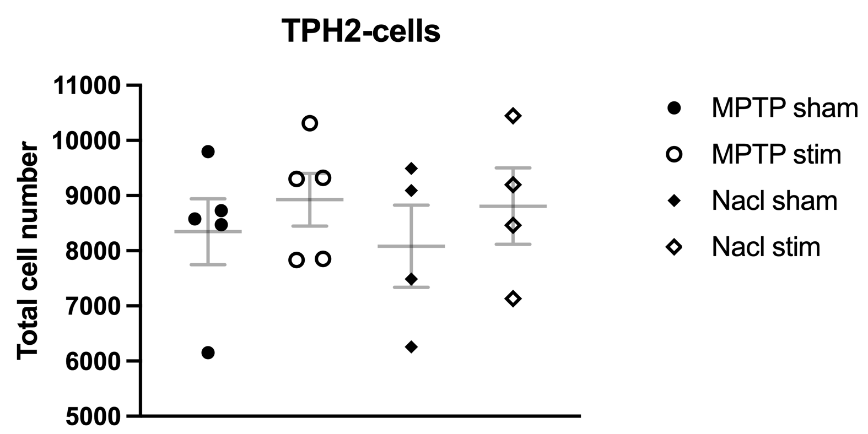

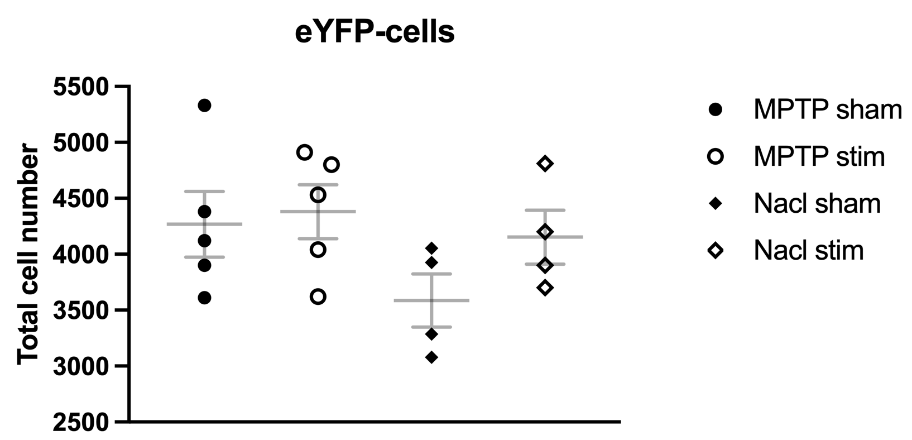


**A**

**B**

Figure 4: **Cell count of TPH2 and eYFP containing cells in the DRN**

Graphs A and B represent the stereological quantification of TPH2 and eYFP containing cells in the DRN of mice, respectively. Statistical analysis did not show any significant difference in number of TPH2 [MPTP-sham: 8345±597 vs MPTP-stim: 8925±477; NaCl-sham: 8083±747; and NaCl-stim: 8810±694, F (3,14) =0.40 p=0.76], and eYFP containing cells [MPTP-sham: 4268±242 vs MPTP-stim: 4380±294; NaCl-sham: 3586±242; and NaCl-stim: 4153±238, F(3,14)= 1.73, p=0.21, Two-way ANOVA, respectively] between groups. Data are presented as mean +/- SEM.
